# Dynamics Insights Into the Gain of Flexibility by Helix-12 in ESR1 as a Mechanism of Resistance to Drugs in Breast Cancer Cell Lines

**DOI:** 10.3389/fmolb.2019.00159

**Published:** 2020-01-24

**Authors:** Abbas Khan, Ashfaq-Ur-Rehman, Muhammad Junaid, Cheng-Dong Li, Shoaib Saleem, Fahad Humayun, Shazia Shamas, Syed Shujait Ali, Zainib Babar, Dong-Qing Wei

**Affiliations:** ^1^State Key Laboratory of Microbial Metabolism, Department of Bioinformatics and Biological Statistics, School of Life Sciences and Biotechnology, Shanghai Jiao Tong University, Shanghai, China; ^2^National Center for Bioinformatics, Quaid-i-Azam University, Islamabad, Pakistan; ^3^Department of Zoology, University of Gujrat, Gujrat, Pakistan; ^4^Centre for Biotechnology and Microbiology, University of Swat, Mingora, Pakistan; ^5^School of Agriculture and Biology, Shanghai Jiao Tong University, Shanghai, China; ^6^Peng Cheng Laboratory, Shenzhen, China; ^7^Joint Laboratory of International Cooperation in Metabolic and Developmental Sciences, Ministry of Education, Shanghai, China

**Keywords:** ESR1 estrogen receptor, mutation, resistance, simulation, molecular docking, molecular dynamics (MD) simulation

## Abstract

Incidents of breast cancer (BC) are on the rise on a daily basis and have proven to be the most prevelant cause of death for women in both developed and developing countries. Among total BC cases diagnosed after menopause, 70% of cases are Estrogen Receptor (ER) positive (ER-positive or ER+). Mutations in the LBD (ligand-binding domain) of the ER have recently been reported to be the major cause of resistance to potent antagonists. In this study, the experimentally reported mutations K303R, E380Q, V392I, S463P, V524E, P535H, P536H, Y537C, Y537N, Y537S, and D538G were analyzed, and the most significant mutations were shortlisted based on multiple analyses. Initial analyses, such as mCSM stability, occluded depth analysis, mCSM-binding affinity, and FoldX energy changes shortlisted only six mutations as being highly resistant. Finally, simulations of force field-based molecular dynamics (MD on wild type (WT) ERα) on six mERα variants (E380Q, S463P, Y537S, Y537C, Y537N, and D538G) were carried out to justify mechanism of the resistance. It was observed that these mutations increased the flexibility of the H12. A bonding analysis suggested that previously reported important residue His524 lost bonding upon mutation. Other parameters, such as PCA (principal component analysis), DCCM (dynamics cross-correlation), and FEL (free energy landscape), verified that the shortlisted mutations affect the H12 helix, which opens up the co-activator binding conformation. These results provide deep insight into the mechanism of relative resistance posed to fulvestrant due to mutations in breast cancer. This study will facilitate further understanding of the important aspects of designing specific and more effective drugs.

## Introduction

Breast cancer (BC) is the primary contributor to a rise in global female mortality rates. It has been reported that 70% of BC cases diagnosed after menopause are Estrogen Receptor (ER) positive (ER+). The human aromatase (HA) enzyme produces estrogens (17-β-estradiol or estrone) (Magistrato et al., 2017) mainly after menopause, and its inactivity increases the level of estrogens in malignant tissues (Liang and Shang, 2013; Sgrignani et al., 2014, 2015, 2016; Magistrato et al., 2017). These hormones have a pro-oncogenic effect by either stimulating cell proliferation or decreasing apoptosis when binding to ERα as an agonist (Liang and Shang, 2013). The endocrine behavior of ER + BC is mainly determined by the deficiency of estrogen, which is caused by the inhibition of downregulators (SERDs), selective modulators of ERα (SERMs), or Human aromatase. Furthermore, as SERMs do leads to ERα ubiquitination and degradation, it also covers the substrate-binding site and alters receptor by changing its conformation (Osborne et al., 2004).

The ligand, which facilitates estrogen activity in several essential physiological processes, regulates ERα, which is a transcription factor and nuclear hormone receptor (Nilsson and Gustafsson, 2011; Lai et al., 2015). The ligand-binding domain (LBD), as well as the DNA-binding domain of ERα (among five separate functional parts), has been determined crystallographically. ERα becomes stable through the binding of either agonists or antagonists under physiological conditions and acts as a dimer. The essential structural element of each LBD monomer is Helix 12 (H12) and can be seen by observing the crystal structures. H12 behaves as a molecular switch between the active and inactive conformation of the receptor. H12 occludes the ligand-binding site on the binding of estrogen to helix packing H3, H5/6, and H11 (Brzozowski et al., 1997; Jordan et al., 2015). It remains stable with ERα's agonist (active) conformation. However, when an antagonist binds, it inhibits H12 from assuming the active conformation, and H12 travels to a groove made by H3 and H5. This relates to antagonistic (inactive) conformation (Joseph et al., 2016). In recent clinical scenarios, there are several effective antagonistic uses of ERα: (i) tamoxifen is a SERM that is active in its metabolites but inactive in peripheral tissues; and (ii) fulvestrant, which is also a SERD, without inactivity regulates ERα, which experiences reduced pharmacokinetic properties (e.g., low water solubility) (Nilsson and Gustafsson, 2011; van Kruchten et al., 2015). In recent decades, the use of tamoxifen by BC patients has reduced the death rate by 25–30%. In ER+ BC patients, 40% get resistance through disease progression and prolonged therapy (Jensen and Jordan, 2003). Recently, Robinson et al. (2013), Toy et al. (2013), Merenbakh-Lamin et al. (2013), and Jeselsohn et al. (2014) reported ERα polymorphisms (mERαs) in the LBD between H9 and H10 (S463P), close to the estrogen binding site (i.e., E380Q) and in the loop that connects H11 and H12 (i.e., L536Q, L536R, Y537C, Y537N, Y537S, and D538G). These polymorphisms occur at a significant rate in relapsed metastatic patients. However, it is rare or absent in untreated patients that have a primary tumor (Robinson et al., 2013; Toy et al., 2013; Jeselsohn et al., 2014). Experimental studies have suggested the possible role of these polymorphisms in inherited resistance to treatments of an endocrine nature (Liang and Shang, 2013) by developing novel features during BC to avoid the use of therapeutics. The reported occurrences of a particular ERα polymorphism vary from case to case, 21–36% of cases in D538G, 5–33% in Y537N, and 13–22% in Y537S, while there are less occurrence of other polymorphisms (Robinson et al., 2013; Toy et al., 2013; Jeselsohn et al., 2014). A double mutant D538G and Y537S was also seen in a few cases (Chandarlapaty et al., 2016). The overall survival time of patients is not dependent on the abundance of these mutations (26 months D538G, 20 months Y537S, and 15 months for double mutant Y537S and D538G) (Chandarlapaty et al., 2016), with the most aggressive isoform being Y537S (De Savi et al., 2015; Lai et al., 2015). A previous study by Pavlin et al. performed a simulation-based study of these mutations, but their analysis utilized only a single simulation tool (Pavlin et al., 2018).

In the multifactorial nature of diseases like cancer, various factors determine the positive, and negative reaction to drugs. The ultimate objective is therefore to investigate the factors directly involved in the development of BC drug resistance and to overcome this problem (Magistrato et al., 2017; Spinello and Magistrato, 2017). So far, computational studies on mERαs and their mechanism have been inadequate to explain K303R, E380Q, V392I, S463P, V524E, P535H, P536H, Y537C, Y537N, Y537S, and D538G polymorphisms that are most repeated isoforms (Delfosse et al., 2012; Robinson et al., 2013; Toy et al., 2013; Fanning et al., 2016; Joseph et al., 2016). It was indicated that these are constitutively active mutants but with a different molecular mechanism (Delfosse et al., 2012; Toy et al., 2013). Other mutants can also have different activation pathways that lead to therapy responses that are mutant dependent and are yet to be studied (Delfosse et al., 2012; Spoerke et al., 2016).

*In silico*, methods to predict structural implications of mutations will be beneficial in understanding mechanisms of drug resistance for quantitative estimation of the phenotypic resistance outcomes (Khan et al., 2019). To systematically understand the effects (protein stability changes, flexibitliy drift, and protein ligand interaction) of these mutations, we performed *in silico* saturation mutagenesis. Additionally, we also assessed the impacts of mutations on the relative sidechain solvent accessibility, depth, and the residue-occluded packing density. Extremely detrimental mutations were selected and analyzed for changes in their interatomic interactions that might explain the destabilizing effects. To explore further the vibrational entropy and enthalpy changes of flexible conformations, we employed an empirical force field-based method, FoldX, a coarse-grained normal mode analysis (NMA)-based elastic network contact model, ENCoM, and a consensus predictor that integrates normal mode approach with graph-based distance matrix in the mutating residue environment. Finally, simulations of force field-based molecular dynamics on wild type (WT) ERα and six variants (E380Q, S463P, Y537S, Y537N, and D538G) were carried out to justify mechanism of the resistance. To cure all types of metastatic BC types, this detailed investigation advocates advancement in precision medicine.

## Materials and Methods

### Comparative Modeling, Quality Assessment, and Model Refinement

A crystallized structure of Estrogen receptor alpha (ESR1) was downloaded from RCSB (**PDB ID 1GWQ**) (Petrella et al., 2011). Structural topology was analyzed for the coordinate's defects. MolProbity (Chen et al., 2010) was used to evaluate the quality of the constructed structure, and atomic conflicts were resolved by energy minimization using the Steepest descent and conjugate gradient algorithms. Using YASARA (Land and Humble, 2018), energy minimization was conducted. The water molecules were completely removed before any further analysis. Pymol was used for visualization (Scientific and San Carlos, 2002). The Fulvestrant structure with accession ID CID802 was obtained from the PubChem database (Kim et al., 2015). The mutant models and sidechains of the mutants were optimized using FoldX (Schymkowitz et al., 2005). Molecular docking of the ESR1 with fulvestrant was performed by using a Schrödinger suite. A glide tool (Friesner et al., 2006) was used for docking of the fulvestrant. Since the structures of fulvestrant and ZB716 are similar, the same protocol using a flexible docking simulation was performed with the Induced Fit protocol (IFD) method as previously reported (Guo et al., 2018). The docking complexes were refined with the protein–ligand interaction refinement tool in the Schrödinger suite. Heirarchial optimization of the complexes that consider the systematic sampling of ligand position, conformation, and orientations along with the proteins residues was performed. All these calculations were performed on the apo structures (wild and mutant) obtained from MD simulation.

### Effects of Mutations on Protein Stability and Interactions

mCSM (http://biosig.unimelb.edu.au/mcsm/) (Pires et al., 2013), SDM (Pandurangan et al., 2017), and FoldX were used to understand the impact of mutations on the thermodynamic stability of the protein. For SDM, mutant-protein models were produced using FoldX, which considers preserved conservation angle laws while identifying the most likely mutant residue sidechain rotamers. To determine the energy fold change upon inducing mutations, FoldX utilizes a linear combination of empirical terms to calculate the effect of mutations on the protein structure in kcal/mol. FoldX uses the following equation to calculate each energy term.

$$\begin{array}{l} \Delta G=a.\Delta G_{vdW}+b.\Delta G_{solH}+c.\Delta G_{solP}+d.\Delta G_{wb}\\ \;+\Delta e.\Delta G_{Hbond}+f.\Delta G_{el}+g.\Delta G_{kon}+h.{T\Delta S}_{mc}\\ \;+\Delta i.{T\Delta S}_{sc}+l.\Delta G_{clash} \end{array}$$

In this expression (*a … l*) are relative weights of the different energy terms used for the free energy calculation. Each term in the above equation is defined in the original manuscript (Schymkowitz et al., 2005).

The effect of mutations on the protein–ligand affinity, ESR1-Fulvestant, was determined by using mCSM-lig (Pires et al., 2016). The mCSM-lig server analyzed only residues within 10Å of the interatomic distance to fulvestrant. The stability changes were further compared with predictions from other computational tools in order to estimate the reliability of the predictions.

### Changes in Vibrational Entropy and Normal Mode Analysis

To evaluate the implications of mutations in flexible conformations on protein stability, we used FoldX, an empirical force field method that computes free energy changes between the protein's native and mutant forms, and an elastic network contact model (ENCoM) (Frappier et al., 2015), a coarse grain NMA strategy that takes into account the nature of the amino acids and aids in calculating vibrational entropy changes upon mutations. We have also used DynaMut (Rodrigues et al., 2018), a protein stability consensus predictor based on ENCoM's predicted vibrational entropy changes and the stabilization changes predicted by mCSM's graph-based signature method.

### Conformational Changes

Conformational changes and their impacts on biophysical properties of the proteins were estimated using SDM (Pandurangan et al., 2017). The interatomic distances between each residue and fulvestrant in the protein-ligand complex were measured and included in the analysis. For all mutations, secondary structure switches in mutants, changes in relative solvent accessibility, residue depth in Å, and residue-occluded packing densities were determined.

### Molecular Dynamics Simulation

In order to estimate the dynamic behavior of fulvestrant at the active site of native and mutant receptors, an all-atoms simulation using an Amber14 package was carried out (Salomon-Ferrer et al., 2013) with the ff14SB force field. For MD simulation, seven systems have been prepared, including a wild type and six complex systems with fulvestrant. Each system was solvated with a rectangular TIP3P water box and neutralized by adding counter ions. The steepest descent minimization method was used for energy minimization followed by conjugate gradient minimization of 3,000 steps. Each system was then gradually heated for 200 ps−300 K. Weak restraints for 2 ns were used to balance each system's density, followed by the constant pressure of 2 ns for system's balance. A constant pressure of using Langevin approach was used (1 atm, 300 K) (Zwanzig, 1973). To evaluate long-range electrostatic interactions the Particle Mesh Ewald (PME) algorithm with default settings in AMBER14 was used (Darden et al., 1993; Essmann et al., 1995). The threshold distances for long-range electrostatic interactions and Van der Waals were set to 10.0 Å, and for hydrogen covalent bonds the SHAKE algorithm was used. (Ryckaert et al., 1977). A total of 700 ns simulations were carried out using pmemd.cuda (Gotz et al., 2012). CPPTRAJ and PYTRAJ packages in Amber 14 were used to evaluate the MD trajectories. We also performed 200 ns simulation for each apo system.

### Unsupervised Clustering of MD Trajectories and Gibbs Free Energy Calculation

Motion in the trajectories from both wild and mutant systems was calculated by using an unsupervised machine-learning technique known as Principle component analysis (PCA) (Pearson, 1901). For this purpose, a CPPTRAJ package in Amber was used. The reference structure was subjected to the translational as well as rotational motions. The positional covariance matrix for atomic coordinates, as well as its eigenvectors, were calculated. The diagonal matrix of eigenvalues was obtained by diagonalizing the matrix with the help of orthogonal coordinate transformation. The eigenvector and its eigenvalue suggested the principal component of the trajectory and highlighted the principal dominant global motion of the structures.

The free energy landscape (FEL) was calculated by using the first two PCs (PC1 and PC2). Deep valleys plot was used to draw and understand the native and metastable states of each system (Hoang et al., 2004). In this study, FEL was calculated using the following equation based on the first two principal components:

$$\begin{array}{l} \Delta G\left(X\right)=-K_{B}TlnP\left(X\right) \end{array}$$

where X suggests the response organizes taken by the primary the two principal components, KB implies the Boltzmann steady, and P(X) is the dispersion of the framework's likelihood on the first two principal components.

### Dynamic Cross-Correlation Map (DCCM) Analysis

Using dynamics cross-correlation maps, the time-subordinate corresponded movements of C-α atom could be plotted. We, therefore, implemented DCCM to comprehend the highly connected movement of the C-alpha atoms when the ligand is bounded.

$$\begin{array}{l} C_{ij}=\frac{<\Delta r_{i}\Delta r_{j}>}{<<{\Delta r_{i}}^{2}><{\Delta r_{j}}^{2}>>} \end{array}$$

The equation above contains various elements of DCCM plots. *D*_*ri*_ and D_rj_ exemplify the vector of displacement of atoms *i* and *j* while **<…>** symbolizes the average trajectory.

### Binding Affinity Calculations

The wild and mutant ESR1 systems free binding energy was figure out by using the script MMPBSA.PY (Hou et al., 2012; Miller et al., 2012; Xu et al., 2013; Sun et al., 2014, 2018; Chen et al., 2016), and these energies were calculated by considering 500s napshots from the MD trajectory. The binding free energy was determined as:

$$\begin{array}{l} \Delta G_{bind}=\Delta G_{complex}-\left[\Delta G_{receptor}+\Delta G_{ligand}\right] \end{array}$$

where Δ*G*_*bind*_ is the absolute free binding energy, and the rest of the parts are the free energy of the complex, the protein, and the ligand. Every segment's free energy was determined by utilizing the given equation:

$$\begin{array}{l} G=G_{bond}+G_{ele}+G_{vdW}+G_{pol}+G_{npol}-TS \end{array}$$

G_bond_, G_ele_ and G_vdW_ indicate interactions among bonded, electrostatic, and van der Waals states, whereas G_pol_ and G_npol_ demonstrate the polar and non-polar binders to the free energy figured by the certain solvent method of the generalized Born (GB) with SASA perceptible to solvents. Ordinary mode investigation determined the entropic commitment of TS.

## Results

### Structure Preparation

**Figure 1:**
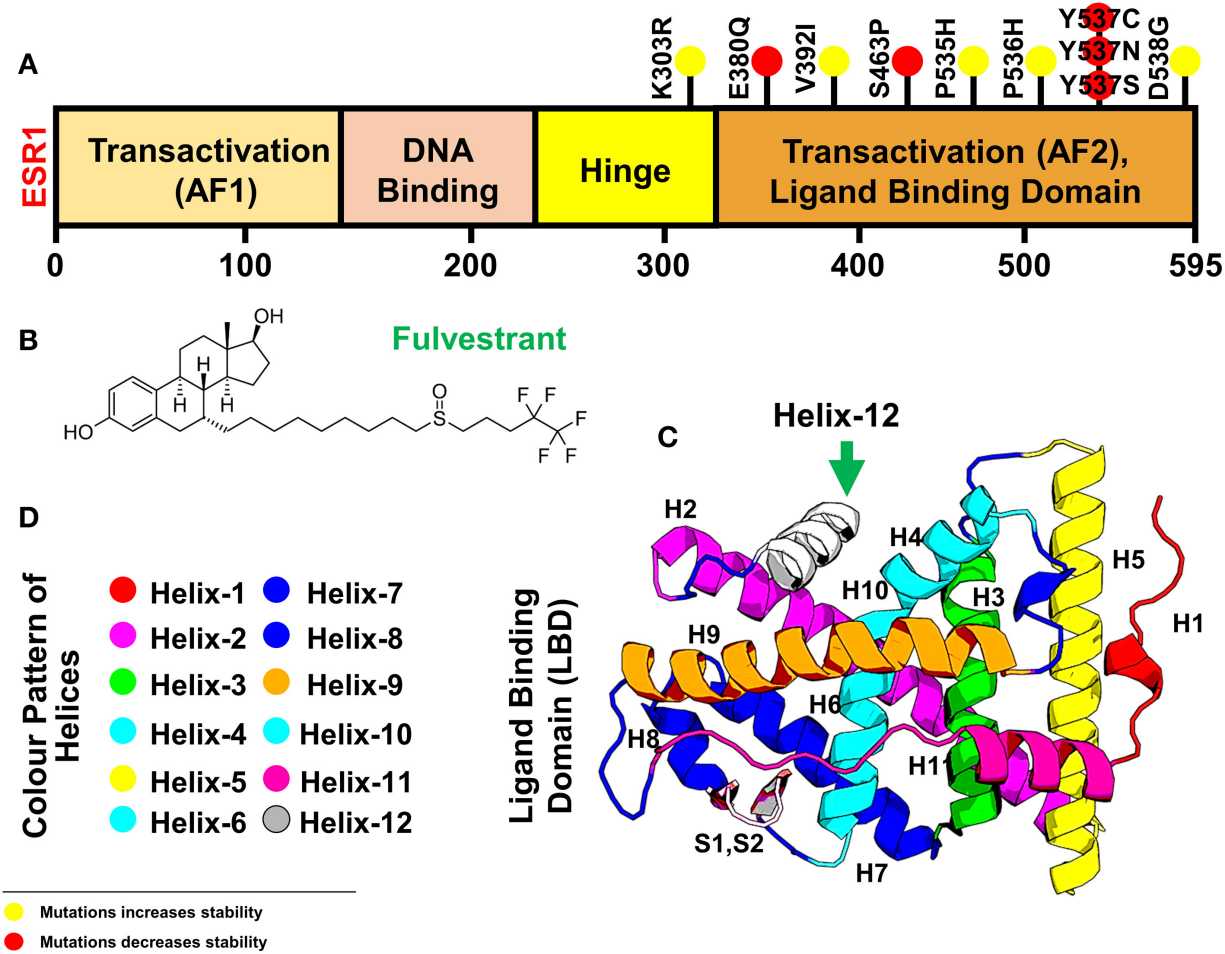
Figure is showing ESR1 and the resistant drug Fulvestrant **(A)** showing different domains [Transactivation domain (AF1), DNA Binding Domain, Hinge Domain, and Transactivation (AF2) or Ligand Binding domain] of ESR1, **(B)** showing the 2D topology of resistant drug Fulvestrant, and **(C)** showing the 3D modeled structure of ESR1 and its different Helices. Helix 12 is highlighted in a green color. **(D)** The color pattern of different Helices shown in **(B)**. The yellow spot represents the resistant mutations with negligible effect on the drug, while the red color spot shows mutations with a significant level of resistance effect.

Structural coordinates of the ligand-binding domain (LBD) of the ESR1 monomer were downloaded from RCSB using PDB ID (1GWQ). An initial visual analysis of the structure revealed some residues were missing while others had defects. The missing residues were identified by comparing them with the primary amino acid sequence of ESR1. Missing residues were added, and structural refinement with YASARA and Fold-X tools was performed. The final structure was subjected to 200 ns simulation to obtain the most stable conformation. Eleven mutations reported by different experimental studies were included as indicated in Table 1. The mutations were spotted in different helices of ESR1, and six mutations were found in Helix 12. Only six mutations E380Q, S463P, Y537S, Y537C, Y537N, and D538G were selected for MD simulation and post-simulation analyses. The selection of these mutations is based on pre-MD simulation analyses, which revealed substantial information about their specificity. In order to get better insight into the mechanism, an initial structure was prepared. The 3D structure of ESR1, its different domains, Helices pattern, and the 2D structure of fulvestrant are shown in Figure 1.

**Table 1:** List of selected mutations and their respective regions (Helix).

**Index**	**Mutation**	**Region**
1.	K303R	Helix-1
2.	E380Q	Helix-12
3.	V392I	Helix-3
4.	S463P	Helix-12
5.	V524E	Helix-11
6.	P535H	Helix-11
7.	P536H	Helix-11
8.	Y537C	Helix-12
9.	Y537N	Helix-12
10.	Y537S	Helix-12
11.	D538G	Helix-12

### Analysis of Free Energy Changes Predicted by Different Computational Tools

Multiple well-implemented algorithms were implied to predict the stability changes associated with each of the mutations in the ESR1 structure. Structure-based stability changes predictors FoldX, mCSM, and SDM were used to calculate effect the possible mutations at each residue position in the LBD of ESR1. The rationale for performing these analyses is to understand how mCSM and SDM, being structure-based predictors of stability change upon mutations and relate to sequence-based methods as well as how vibrational entropy changes in normal mode perturbations.

### Mutations-Stability Correlation Analysis

mCSM uses graph-based signatures, where the primary feature is distances between different atoms. It also uses the common pharmacophoric features and converts it into digits where its mutant pharmacophore count is compared with the wild one. Wild type and mutant residues are represented as pharmacophore frequency vectors. These feature vectors are appended to experimentally important thermodynamics features, such as pH, solvent accessibility, and temperature. Herein, using mCSM, stability changes upon mutations were calculated, and average changes ranging from 0.823 to −3.033 kcal/mol was reported. Mutations, such as E380Q with stability fold change −1.192 kcal/mol and S463P with stability fold change of −0.689 kcal/mol, Y537S, Y537N, and Y537C with stability fold changes of −1.899, −1.66, and −0.566 kcal/mol, respectively, were found to be primarily affected in the highest fold with a destabilizing effect. In addition, mutation D538G with stability change of −0.545 kcal/mol was also clustered as destabilizing mutants. Mutations E380Q and S463P lie near or in the active site (Helix 3 and Helix 11), thus posing high level of resistance to the drug.

On the other hand, mutations Y537S, Y537N, Y537C, and D538G are spotted in Helix-12, whose flexibility is affected by these mutations and thus pose significant flexibility drift. It has been previously reported that the flexibility and replacement of Helix-12 can cause major destruction on the binding of ligands in the active site. The active site residues lie on Helix-3 and Helix-11, which is affected by the motion of Helix-12. According to the results, the remaining mutations, such K303R, V392I, V524E, P535H, and P536H, produce the opposite effect (i.e., stability) and do not induce major changes in affinity or reported to be least influenced. DynaMut, DUET, CUPSAT, and I-mutant also rationalized the destabilizing effects of the six mutations E380Q, S463P, Y537S, Y537C, Y537N, and D538G. All the stability results predicted by different servers are given in Table 2.

**Table 2:** The table shows the stability results predicted by different servers and softwares (DUET, ENCoM, DynaMut, mCSM, SDM, and FoldX).

**Index**	**Mutation**	**DUET**	**ΔΔG ENCoM**	**ΔΔG DynaMut**	**mCSM ΔΔG**	**SDM ΔΔG**	**FoldX**	**Outcome**
1.	K303R	0.116	−0.025	−0.217	−0.719	−0.17	−0.03	Stabilizing
2.	E380Q	−1.192	0.03	0.416	−1.482	−0.4	−0.29	Destabilizing
3.	D538G	−0.445	0.028	0.265	0.008	−0.54	0.92	Destabilizing
4.	Y537S	−1.899	−0.027	−0.095	−0.215	−0.39	3.22	Destabilizing
5.	Y537N	−1.369	−0.809	−0.077	−0.315	−1.66	2.41	Destabilizing
6.	H524E	−0.243	−0.087	−0.198	−0.73	−0.11	2.02	Destabilizing
7.	V392I	−0.431	−0.6	−1.726	−0.264	0.16	−0.86	Stabilizing
8.	S463P	−0.495	−0.606	−1.188	−0.689	−0.09	3.69	Destabilizing
9.	P535H	−0.036	−0.08	−0.056	−0.312	−1.1	1.65	Destabilizing
10.	L536H	−0.044	−0.413	−0.174	−0.788	−0.77	0.16	Stabilizing
11.	Y537C	−0.566	−0.559	0.076	−0.164	0.27	2.22	Destabilizing

### Impact of Mutations on Flexible Conformations and Changes in Vibrational Entropy (ΔS)

FoldX calculated the stability changes between the wild type and each mutant in the lowest energy conformation. It optimizes the sidechain rotamers of the mutant residues to attain a low energy state and calculates the change in free energy between the states. It can be seen from Table 2 that the mCSM suggested mutations E380Q, S463P, Y537S, Y537C, Y537N, and D538G are more likely to affect the energy changes in the higher fold than the others. It can be confirmed that mutations in helix-12 are primarily the major contributor in the energy changes profile.

**Figure 2:**
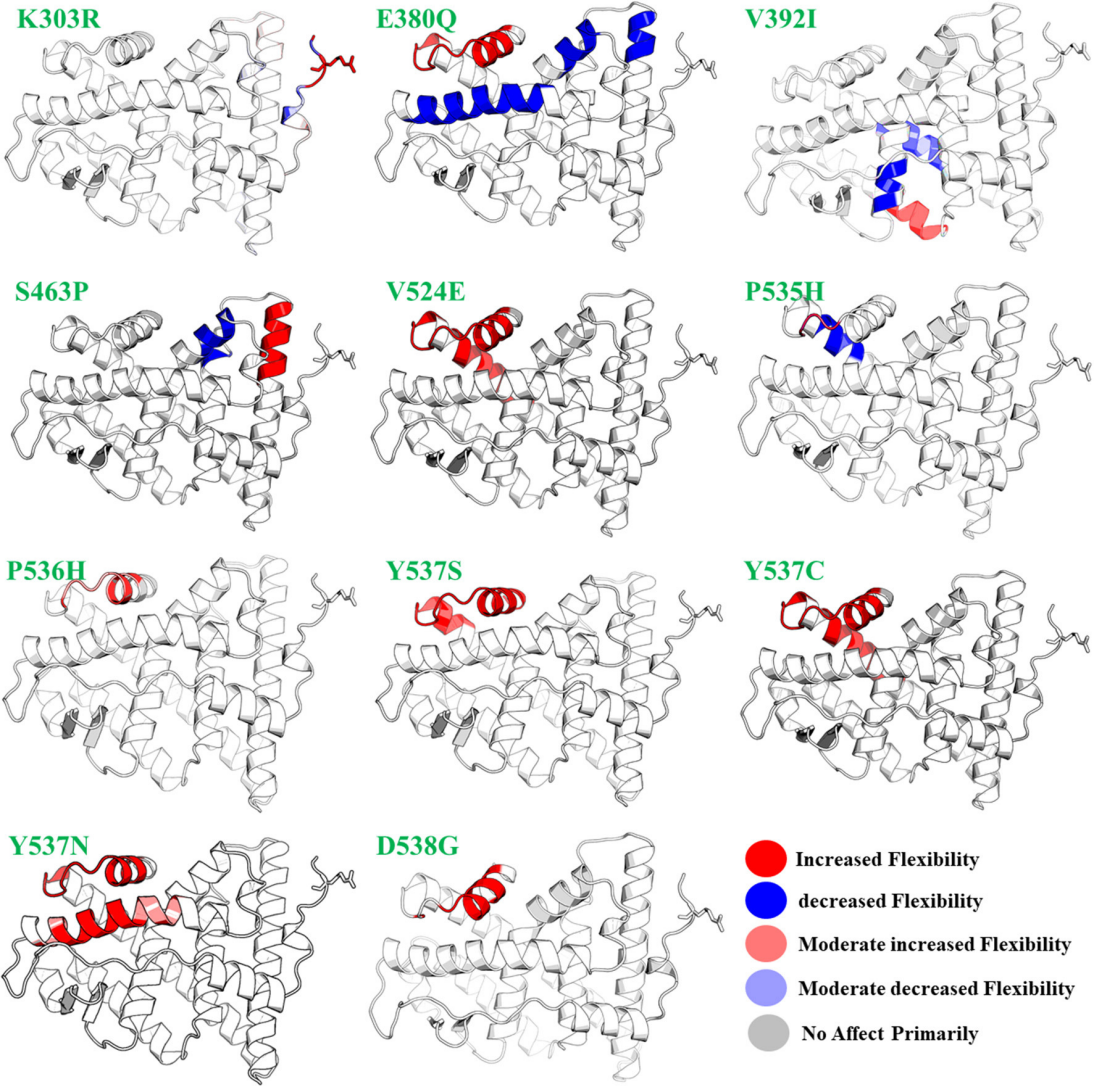
The effect of mutations on the flexibility of different residues. Different colors represent different levels of flexibility.

On the other hand, fully flexible conformers of the mutants were sampled to compute the difference in vibrational entropy(ΔS) between the mutants and wild type. The average vibrational entropy change was observed from −0.038 to 1.011. All the calculations were carried out in kcal/mol.K^−1^. The maximum vibrational entropy (ΔS) changes were induced by mutation Y537N, followed by S463P, Y537C, L536H, D538G, Y537S, and E380Q. It can be seen in Figure 2 that the six mutations Y537N, S463P, Y537C, D538G, Y537S, and E380Q induced higher flexibility than those of the others. These results, along with the other, i.e., stability changes, clearly pointing out the importance of these six mutations. It has been previously reported that these mutations specifically in the Helix-12 pose major resistance to treatment in breast cancer. It can be seen that mutations D538G, Y537N, Y537S, and Y537C in helix 12 destabilizes the protein conformation by inducing significant flexibility drift.

Furthermore, mutations, such as E380Q and S463P, increase the residual rigidity in some helices while alternative inducing flexibility in some residues. These changes in flexibility (red) and rigidity (blue) are mapped onto the corresponding protein structure and presented in Figure 2. In order to get further insight into the phenomena, we conducted a simulation analysis of each apo system for a total of 200 ns. The results suggested that the mutations induced stability and conformational changes in the structure of protein. The root mean square fluctuation (RMSF) was calculated to confirm the flexibles regions and residual flexibility changes upon mutations. It was noticed that mutations, such as D538G, Y537N, Y537S, and Y537C, in helix 12 expand its motion. The results of RMSD, RMSF, b-factor, radius of gyration, and cross-correlation analysis are given in Figures S1–S5. These results suggest that the major fluctuation and destabilization effect was caused by E380Q, S463P, Y537S, Y537C, Y537N, and D538G. Thus, it can be inferred that the antagonist and co-activator binding conformation is stabilized by these mutations. When compared to the wild type apo helix-12, this region of the six mutations E380Q, S463P, Y537S, Y537C, Y537N, and D538G was found to have greater flexibility (RMSF Figure S2).

To give further insight into conformational changes induced by these mutations, secondary structure switches in mutants, changes in relative solvent accessibility, residue depth in Å, and residue-occluded packing densities were determined. From the maximum destabilizing mutations, increases in RSA, residue-occluded packing densities, and decrease in depth were observed and are tabulated in Table S1.

Herein, using mCSM-lig, affinity changes upon mutations were calculated, and average changes ranged from 0.823 to −3.033 kcal/mol was reported. Mutations, such as E380Q with affinity fold change −1.399 kcal/mol, S463P with affinity fold change of −1.305 kcal/mol, Y537S, Y537N, and Y537C with affinity fold changes of −1.098, −0.878, and −0.931 kcal/mol, were found to be primarily affected in highest fold with destabilizing effect. In addition, mutation D538G with an affinity fold change of −0.909 kcal/mol was also clustered in destabilizing mutants. Mutations E380Q, S463P, Y537S, Y537N, Y537C, and D538G were spotted in Helix-12 whose flexibility is affected by these mutations and thus poses a significant flexibility drift. It has been previously reported that the flexibility and replacement of Helix-12 can cause major destruction on the binding of ligands in the active site (Kuang et al., 2018). The active site residues lie on Helix-3 and Helix-11, which is affected by the motion of Helix-12. According to mCSM results, the remaining mutations, such K303R, V392I, V524E, P535H, and P536H, do not induce significant changes in affinity or reported to be least influenced. The results obtained from these analyses are tabulated in Table 3.

**Table 3:** FoldX energy changes and mCSM-lig ligand binding affinity fold change prediction upon mutation in ESR1.

**Index**	**Mutations**	**ΔΔS ENCoM**	**Affinity fold change**	**Docking** **score**
1.	Wild	00	0.00	−10.5
2.	K303R	0.032	−0.445	−9.54
3.	E380Q	0.338	−1.399	−6.27
4.	D538G	0.355	−0.909	−8.97
5.	Y537S	0.340	−1.098	−8.63
6.	Y537N	1.011	−0.878	−8.87
7.	H524E	0.109	−0.429	−8.7
8.	V392I	0.25	0.154	−10.5
9.	S463P	0.758	−1.305	−9.54
10.	P535H	0.1	0.481	−6.27
11.	L536H	0.517	0.444	−8.97
12.	Y537C	0.699	−0.931	−8.63

*For the wild type, the experimental concentration already reported 0.138 nM was used*.

### Interaction Analysis

**Figure 3:**
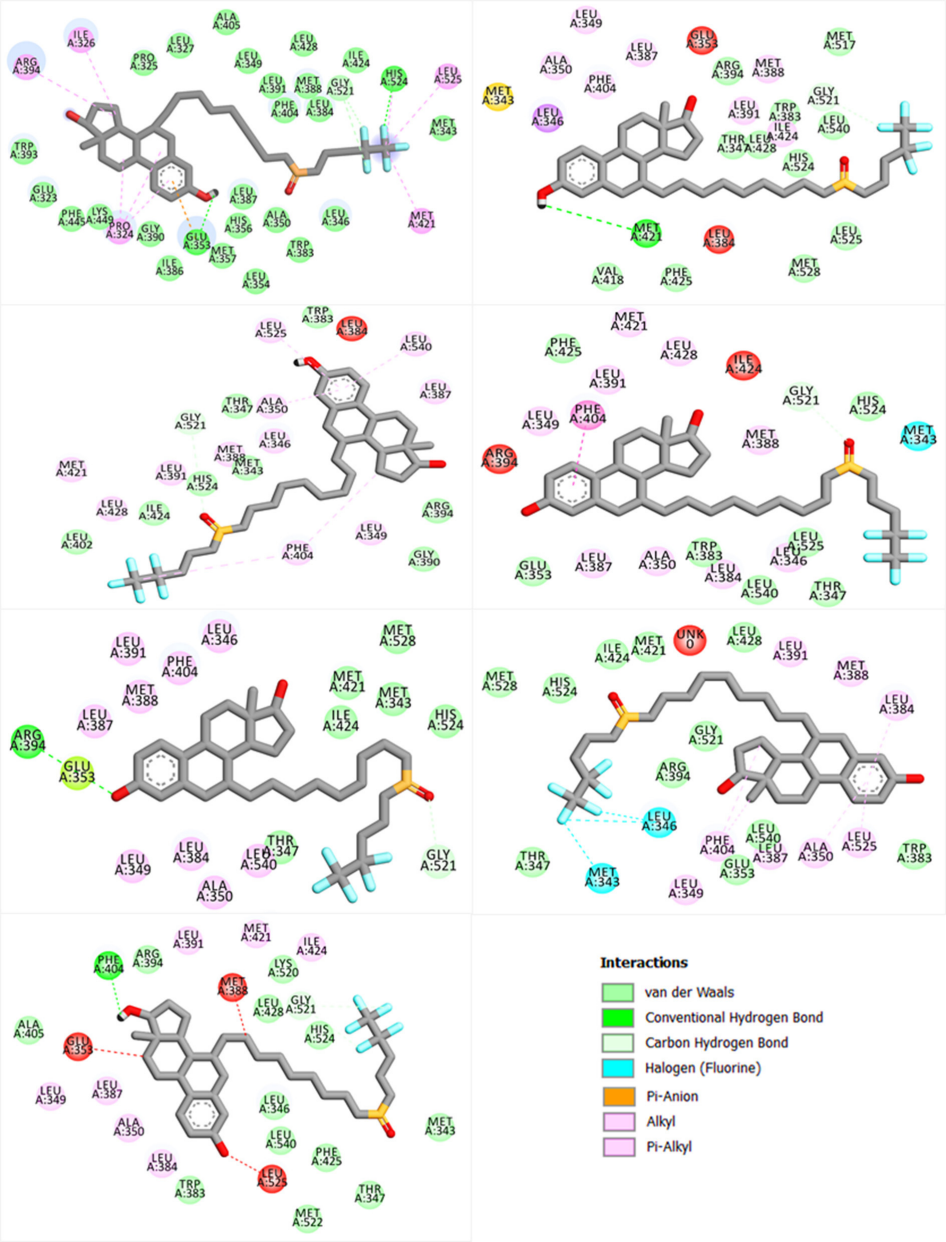
Showing the bonding pattern of fulvestrant with ESR1 (wild and mutants). The interaction legend is also given in the bottom.

Important residues, such as Glu353, Arg394, and His524, are important for antagonist activity. Upon docking with a wild type and mutants, these residues were considered for the bonding analysis. In case of wild type as given in Figure 3, a strong hydrogen bond with His524 and Glu353 can be easily seen, while, in case of mutations, such bonds are especially absent with His524. Thus, we speculate that these mutations also disturb the bonding pattern of fulvestrant with ESR1. Furthermore, other hydrophobic and electrostatic interactions are also formed and lost. It is also important that targeting Cys530 residue could help in resolving the resistance posed by these mutations (Furman et al., 2019). The number of hydrogen bonds before and after the MD simulation and their lengths are given in Table S2.

### Dynamics and Conformational Transition of Wild and Mutated ESR1

**Figure 4:**
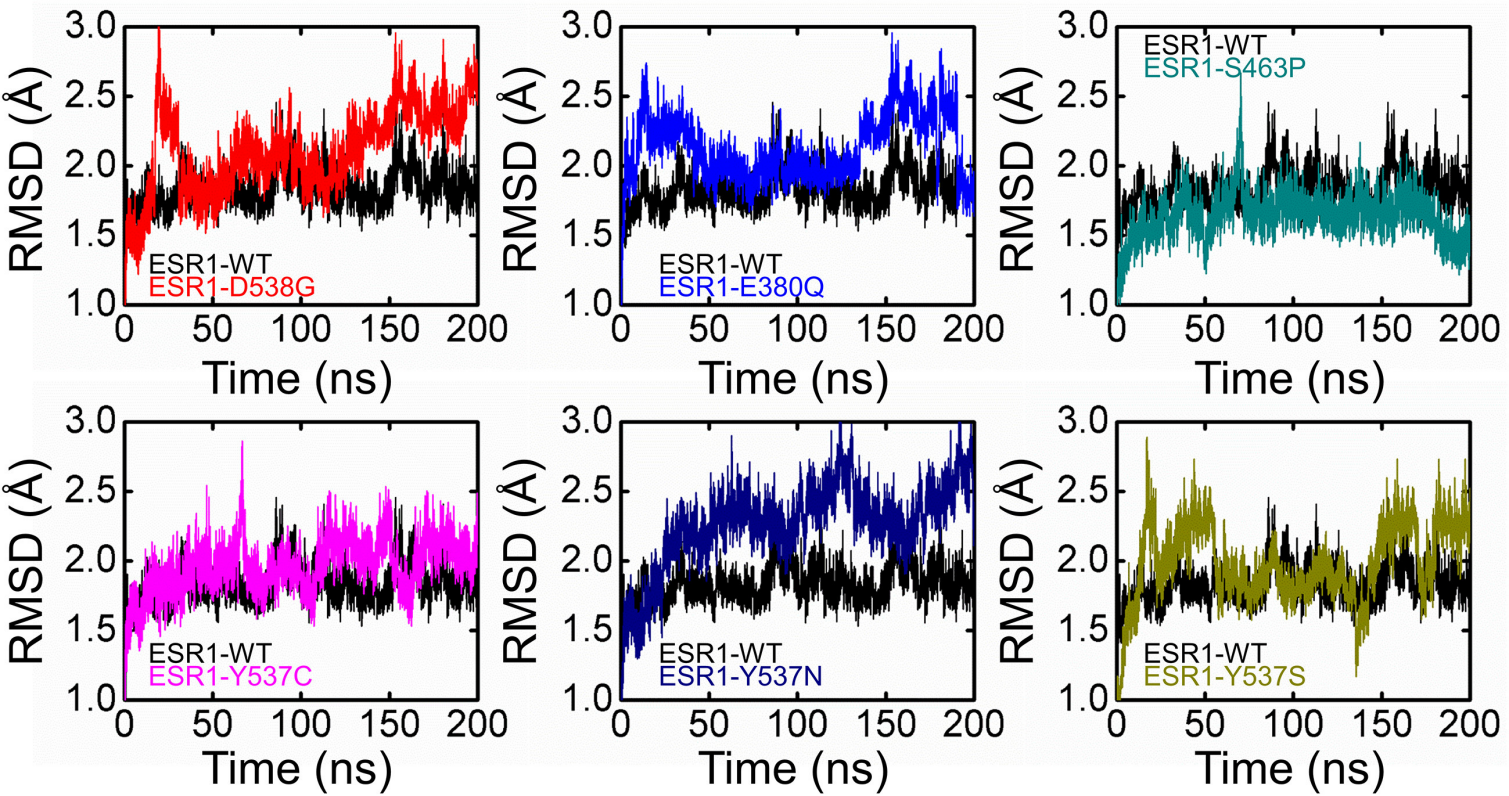
Root Mean square deviation of all the systems compared with the wild type. The black color is showing the wild while the rest of the colors represent the mutant systems.

Dynamics features of all the systems, including RMSD, RMSF, per-residue RMSF analysis, radius of gyration, and distances of important atoms/residues of both wild and mutant systems were calculated after a total of 1,400 ns of simulation time. Different effects of these mutations differentially affected the dynamics of these systems. While calculating RMSD, it was found that the wild system attained the steady-state soon after reaching 10 ns. While compared to the wild type, these systems (D538G, E380Q, and S463P) and the RMSD values are relatively high, which is due to the induction of mutation in the H12 helix, and thus affect the stability of the protein by targeting the specific residues. It has been discussed in our results that these mutations, compared to the other Y537S/N/C, did not affect the protein significantly. On the other hand, mutations induced at Y537S, Y537C, and Y537N greatly affected the protein dynamics and stability. It can be seen that the mutations induced in the H12 at Y537 residue posses higher RMSD than the wild system, which, as has been discussed in the above results, means that these mutations significantly affect the protein dynamics, and thus stability is mostly influenced. These results are consistent with the results predicted by different methods that the mutation Y537S/N/C affects the protein in a higher fold as compared to the other three. Previous studies based on other antagonists also suggested that Y537S/N/C is less stable than the others. These mutations are previously prioritized to be primarily treated for the successful treatment of breast cancer. Thus, the results we obtained are consistent with the results predicted in other algorithms and pervious studies (Jeselsohn et al., 2015; Kuang et al., 2018). All the RMSDs are given in Figure 4.

**Figure 5:**
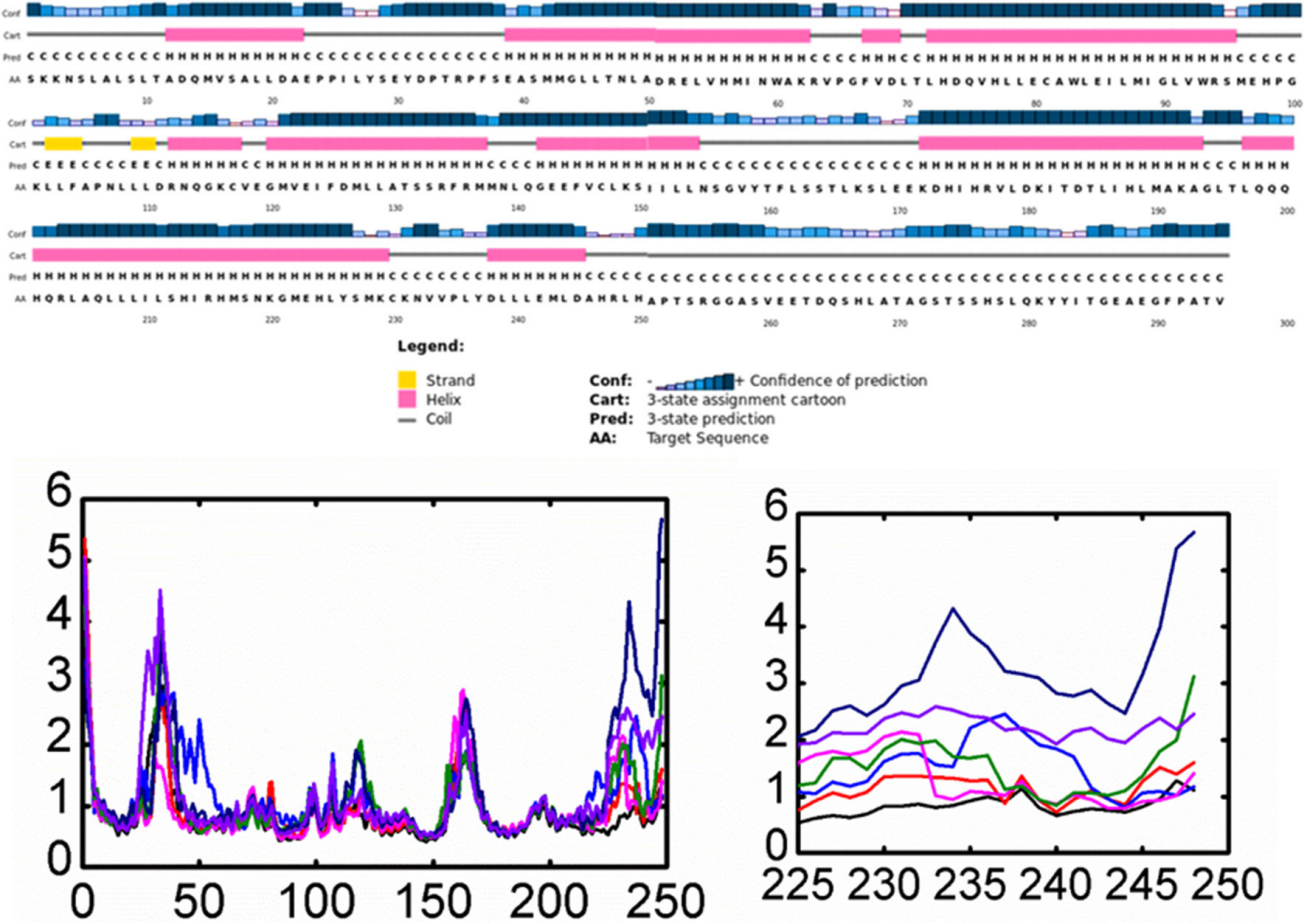
Root Mean square fluctuation of all the systems compared with the wild type. The left panel is showing the RMSF and the complete structure of ESR1. The right panel is showing the fluctuation of Helix-12 which is confirming the flexibility drift caused by the mutations. Each system is shown in a different color.

Furthermore, residual fluctuations and fluctuation of the H12 helix residues were calculated as RMSF and per-residue RMSF. Analyses of the root mean square fluctuations (RMSFs) by residue in the wild and mutant complexes were compared. It was found that fluctuation in the H1 helix was observed due to its continues loop structures and accommodated by few helix residues. During the simulation time, fluctuation in these residues was observed to be higher. We also observed high dynamic activity in a loop preceding H9 helix (residues 160–166). The most important feature of these wild and complex systems was understanding the flexibility of H12 helix in all the systems. It has been previously shown that hydrogen bonds formed by His524 residue with the antagonist could reduce the motion of H11 and H12 helices and thus maintain the antagonist conformation of the protein and avoid the binding of co-activator which could lead to the reduced activity of this co-complex in breast cancer. Here, a per-residue RMSF analysis was correlated with the mutations, and the RMSF of each residue lies in the H12 helix was calculated. As given in Figure 5, it can be easily observed that the mutation affected the residual flexibility of the H12 helix in a higher fold when compared with the wild system. It can be seen that residual flexibility of each of these residues in H12 helix is higher than that of the wild system. The flexibility of H12 increases with the simulation time. Thus, these mutations substantially affected this helix.

Experimental studies proposed the mechanism that the movement of H12 is mainly responsible for the conformational transition between agonist and antagonist and the binding of a co-activator. Thus, the loss of important hydrogen and other bonds with the enhanced flexibility reduces the efficacy of the antagonist and thus fails to restrain the movement of the H12 helix and halt the binding of co-activator to the ESR1. Therefore, the increased flexibility favors the binding of a co-activator by supporting the agonist conformation, which was already reported in the previous study. These hotspot mutations, specifically Y537S, Y537C, and Y537N, affect the flexibility of the H12 in the highest fold and are shown in Figure 5 (right panel). Previous results also suggested that these mutations primarily destabilizes the binding of antagonists and reduces the interactions with the H12 by enhancing the conformational transition. Our results also support the early results reported by different studies: the role of H12 in terms of its flexibility is the primary cause of resistance (Merenbakh-Lamin et al., 2013; Robinson et al., 2013; Toy et al., 2013), and Y537S/C/N are significantly involved in the resistance to fulvestrant (O'Leary et al., 2018).

**Figure 6:**
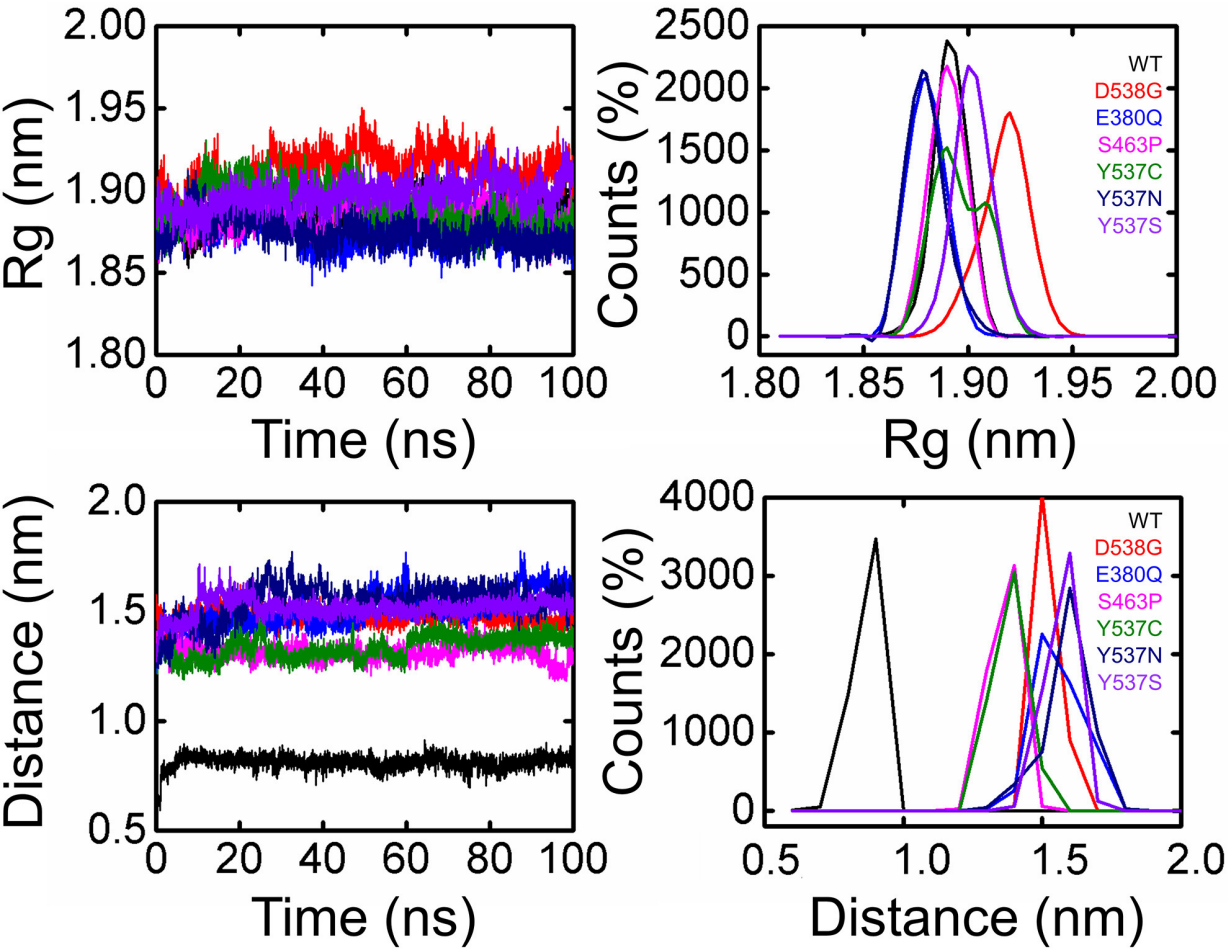
Radius of gyration and Distance of 5,000 snapshots obtained from each system are shown in different color.

To further demonstrate the mechanism of resistance posed by the H12 helix, we calculated the distance between the ligand and residue His524, an essential parameter in determining the role of agonist and antagonist conformation stabilization. As given in Figure 6, the distance between the ligand and His524 is minimal in the wild system, while in the other systems with mutations this distance was found to be increased range from 0.15 to 0.18 nm in different mutations. The average distance in the mutated systems was ~0.15 nm. Hence, the movement of H11 and H12 is highly correlated, and we speculate that the loss of important interactions due to the flexibility could allosterically affect the H11. It has also been previously shown that this is due to the rotation of His524 from a –guache to +guache conformation. However, the bond between Glu339 of H3 and Lys531 of H11 ties together and avoid the unwinding of the protein. Here, the fluctuation of bond distance between the ligand and His524 is also an important factor in determining the stability of each conformation (agonist and antagonist) because a shift in the orientation between agonist and antagonist has been previously reported to be associated with interaction with His524 too. The radius of gyration is given in Figure 5.

### Trajectories Transitions and Dynamic Motions

**Figure 7:**
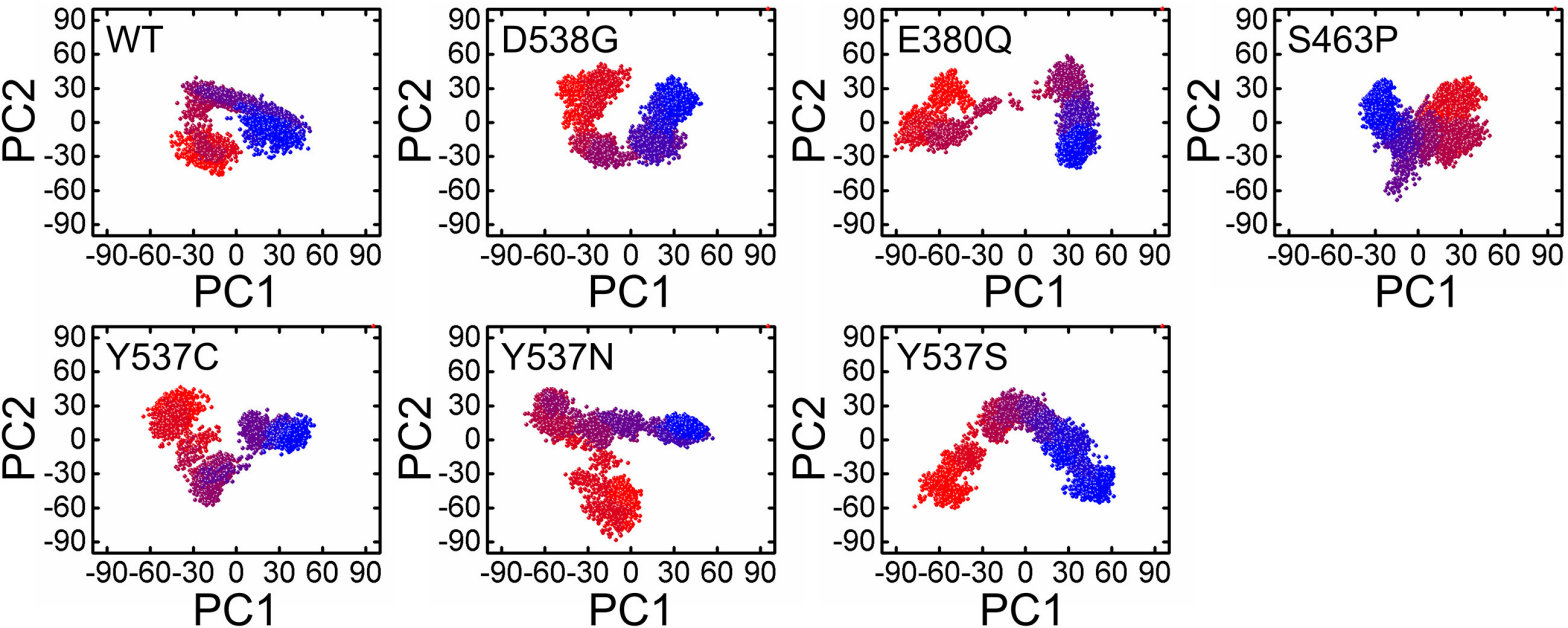
Principle component analysis of 5000 snapshopts obtained from MD simulation of all the systems.

A PCA (Principal component analysis) was applied to study and used to evaluate the distinct protein conformational states in a principal component (PC) phase space during the molecular dynamics simulations. Trajectories were projected onto a two-dimensional subspace using the first three eigenvectors, i.e., PC1, PC2, and PC3, to understand the conformational transitions of the complexes. Figure 7 shows that all the complexes attained two conformational states on the subspace differently colored (Figure 7). The conjoined distributions of the principal components of the complexes discovered that the energetically unstable conformational state blue neared convergence and attaining a stable conformational state red color. Consequently, different periodic jumps are required for the transition of different conformations in mutants.

**Figure 8:**
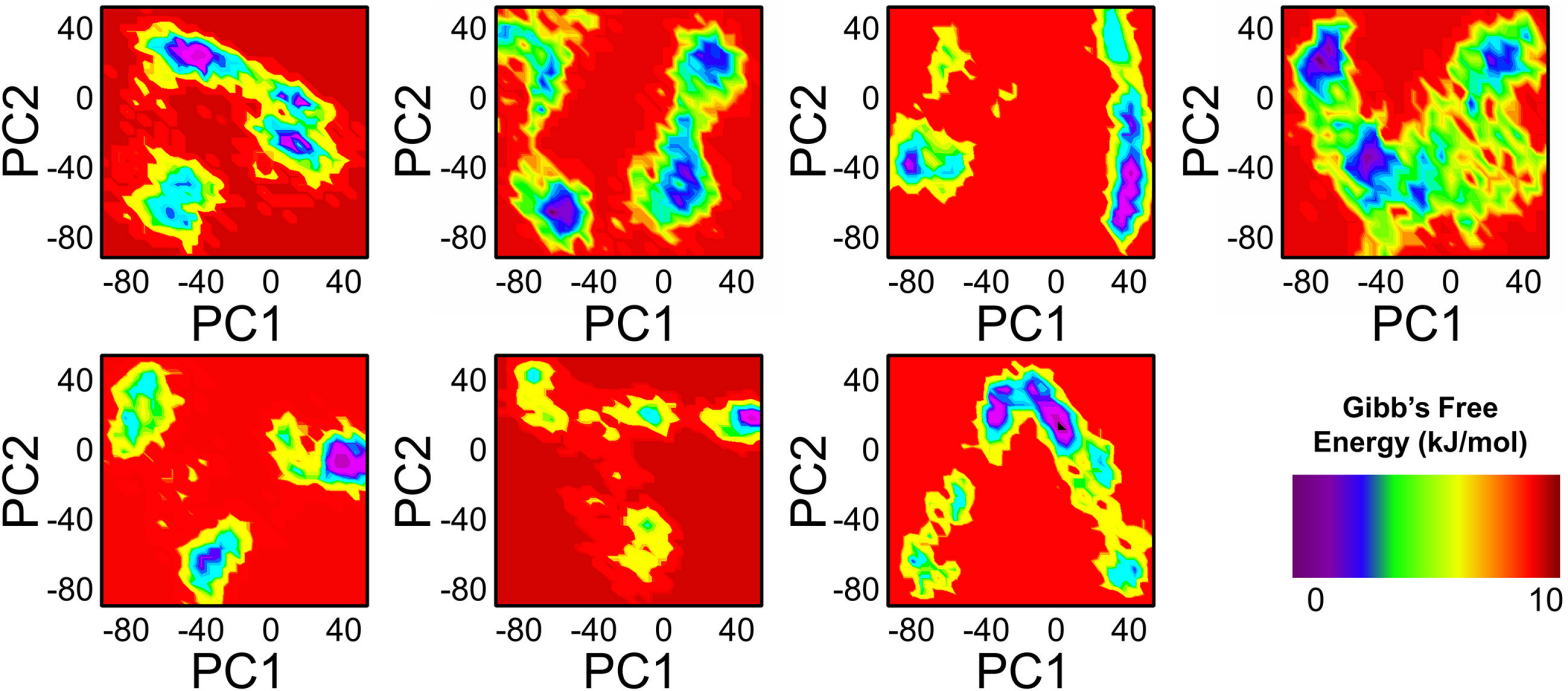
FEL of PC1 and PC2 obtained from MD simulation of all the systems.

To further understand the transition mechanism of mutants and wild complexes from metastable to native states, eigenvectors (the first two) were used to calculate and to plot the free energy landscape (FEL) of the 200 ns trajectory time. Low energy states were extracted to understand structural evolution. As shown in Figure 8, the lowest Gibbs energy states are highlighted using a dark purple color, while the numbers represent the positions of the structural coordinates sampled from that locus on the FEL plot. The FEL plot showed that the wild type attained four different energy states (three metastable and one native). On the other hand, mutations D538G, S463P, E380Q, and Y537S were found to have three states, including one native and two metastable states. Mutations Y537C and Y537N probably formed two metastable states only. The result indicates the frequent transition of the conformations in the mutants compared to wild type. Mutations adopted multiple metastable states during their structural evolution in MD simulations and were separated by low- and high-energy barriers, respectively. Interestingly, mutations like Y537C, Y537N, and Y537S were observed in its profoundly transition state by observing the RMSD plot.

**Figure 9:**
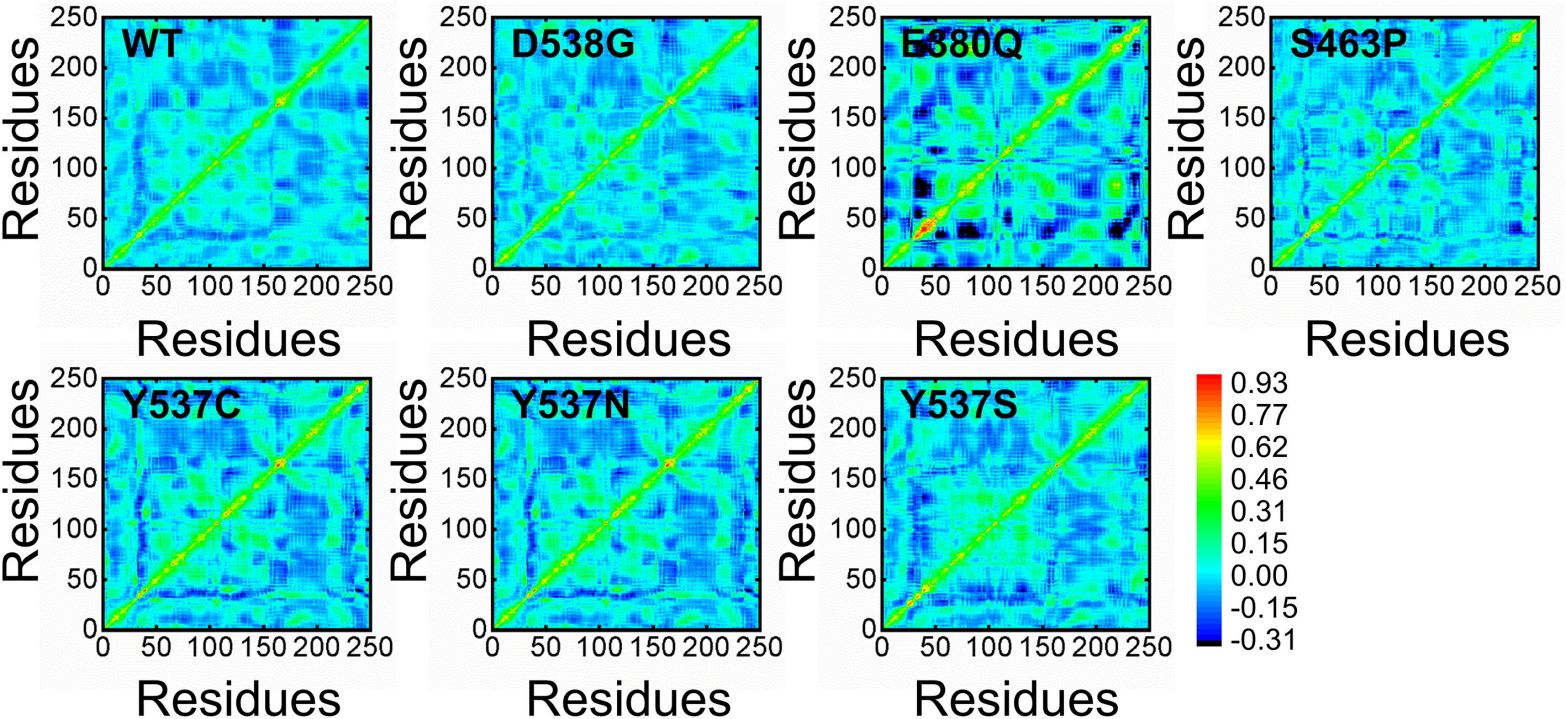
Distance cross-correlation matrix of both wild and mutant systems.

To further examine the residue's correlative motions, trajectories were subjected to a dynamics cross-correlation analysis. A diverse pattern of correlations was observed in all the systems. The atomic displacement in mutants was observed to be high when compared to wild. It can be seen that highly atomic displacement of H12 in Y537C/N/S is experienced, while in the case of E380Q and S463P these motions are observed in multiple atoms of different residues. The results given in Figure 9 clearly show that all mutants display different correlated motions than the wild complex.

## Discussion

In practice, ER proved to be the prime target for BC therapy, but poor response or complete resistance developes during the course of treatment, making treatment a grim challenge and BC lethal. Comprehension of these mechanisms at a cellular and genetic level is of paramount importance to evade this muddle and come up with an effective treatment. Fulvestrant is a potent antagonist, and it has been characterized to reduce the burden of breast cancer. The resistance to fulvestrant, caused by genetic aberrations in ESR1, has been reported (Shi et al., 2014; Akhmetova et al., 2015; Khan et al., 2018), but the molecular mechanism subsequently leading to resistance has not yet been elucidated. Here, we used a logical approach to understand the mechanism's underlying resistance to targetting ER, using structure-guided approaches. The present examination gives insight into the mechanism behind the fulvestrant resistance, which could help in designing new anti-BC drugs. We adopted an extensive computational procedure to unveil the molecular mechanisms of resistance offered by ESR1 mutations to fulvestrant. The activity of a native protein is affected by an aberration that can occur anywhere, not only in the residues of the active site. Previous studies demonstrated that such changes have a remarkable impact on the structure and action of fulvestrant. In the present investigation, the Y537S, Y537C, and Y537N mutations significantly influence the activity of the fulvestrant drug. Using multiple servers, such as mCSM, SDM, DUET and many others, the servers reported that six mutations have significantly affected the activity of the fulvestrant. Also, a molecular dynamics simulation revealed that the structural stability and flexibility directly correlated to the mutation. All these analyses suggested that the flexibility of H12 could open up the co-activator confirmation due to enhanced flexibility. Differences in the docking and free energies also clarified the distortion caused by these mutations. Furthermore, the principal component analysis, free energy landscape, and dynamics cross-correlation analysis also clarified the dominant motions, native and metastable state, and correlated motions in ESR1 (wild and mutant systems). These methods have previously been used by different studies to understand protein dynamics, mechanim of resistance, and drug interaction (Du et al., 2016, 2019; Yang et al., 2018).

In conclusion, we quantified the impact of reported mutations K303R, E380Q, V392I, S463P, V524E, P535H, P536H, Y537C, Y537N, Y537S, and D538G in the activity of fulvestrant. This study clarified how these mutations alter structural properties, binding affinity, stability, and resistance in breast cancer treatment. Our results provide further understanding into the factors associated with drug resistance in breast cancer cell lines and thus provide a useful pathway for the development of new medications for treatment of breast cancer.

## Data Availability Statement

The raw data supporting the conclusions of this article will be made available by the authors, without undue reservation, to any qualified researcher.

## Author Contributions

AK conceptualized the methodology. AK, A-U-R, and MJ did the analysis. FH, SSa, SA, C-DL, SSh, and D-QW wrote the manuscript. AK and ZB revised the manuscript and performed all the revised analyses. D-QW supervised the study. All authors approved the manuscript.

### Conflict of Interest

The authors declare that the research was conducted in the absence of any commercial or financial relationships that could be construed as a potential conflict of interest.

## Supplementary Material

The Supplementary Material for this article can be found online at:

https://www.frontiersin.org/articles/10.3389/fmolb.2019.00159/full#supplementary-material
