# Supplementary material for: Dynamics Insights Into the Gain of Flexibility by Helix-12 in ESR1 as a Mechanism of Resistance to Drugs in Breast Cancer Cell Lines
Source: Front Mol Biosci. 2020 Jan 24;6:159. doi: 10.3389/fmolb.2019.00159 (PMC6992541; doi:10.3389/fmolb.2019.00159)
Supplement: Supplementary file 1 [file Data_Sheet_1.docx]

**Supplementary Material**

**Dynamics insights into the gain of flexibility by Helix-12 in ESR1 as a mechanism of resistance to drugs in breast cancer cell lines**

Abbas Khan^1^, Ashfaq-Ur-Rehman^1^, Muhammad Junaid^1^, Cheng-Dong Li^1^, Shoaib Saleem^2^, Fahad Humayun^1^, Shazia Shamas^3^, Syed Shujait Ali^4^, Zainib Babar^7^, Dong-Qing Wei^1,5,6^

^1^State Key Lab of Microbial Metabolism, Department of Bioinformatics and Biological Statistics, School of Life Sciences and Biotechnology, Shanghai Jiao Tong University, Shanghai, 200240, China.

^2^National Center for Bioinformatics, Quaid-i-Azam University, 45320 Islamabad Pakistan.

^3^Department of Zoology, University of Gujrat, Pakistan

^4^Centre for Biotechnology and Microbiology, University of Swat, Swat, Khyber Pakhtunkhwa, Pakistan.

^5^Peng Cheng Laboratory, Vanke Cloud City Phase I Building 8, Xili Street, Nashan District, Shenzhen, Guangdong, 518055, P.R China.

^6^Joint Laboratory of International Cooperation in Metabolic and Developmental Sciences, Ministry of Education, P.R China.

^7^School of Agriculture and Biology, Shanghai Jiao Tong University, Shanghai, 200240, China.

Corresponding author

Dong-Qing Wei

[dqwei@sjtu.edu.cn](mailto:dqwei@sjtu.edu.cn)


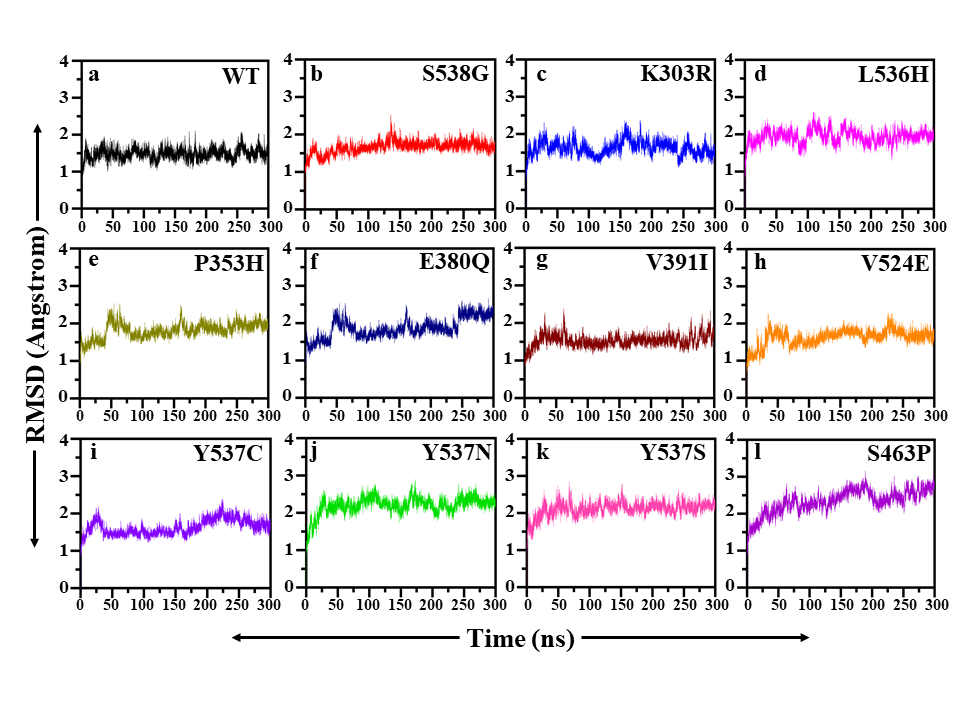
**Figure S1:** Showing the Root mean square deviation (RMSD) of all the apo systems (Wild and Mutant).


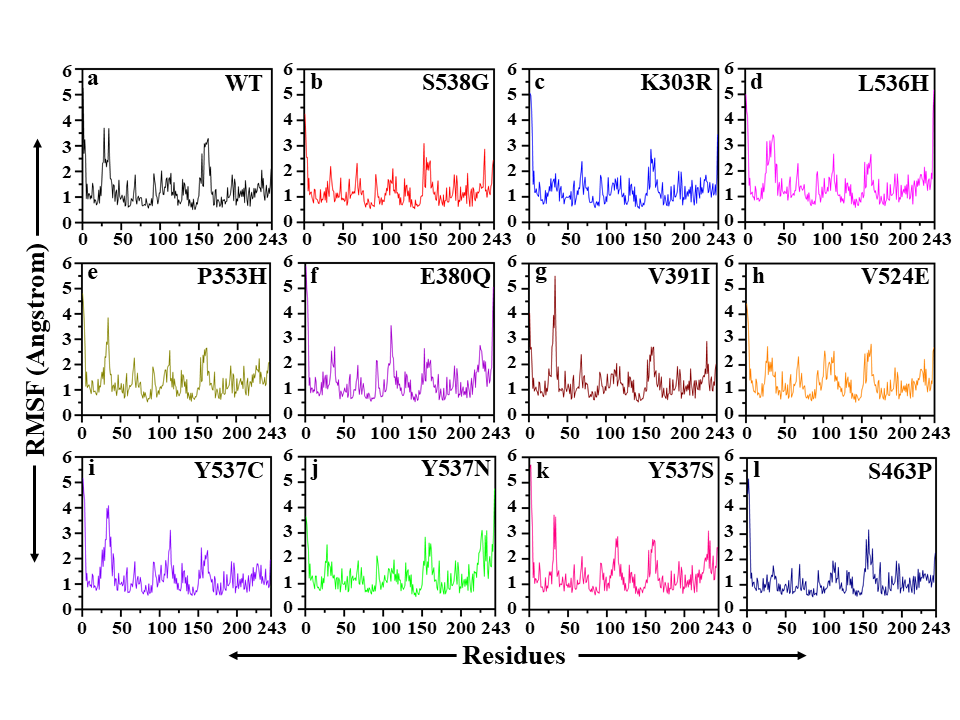


**Figure S2:** Showing the Root mean square fluctuation (RMSF) of all the apo systems (Wild and Mutant).


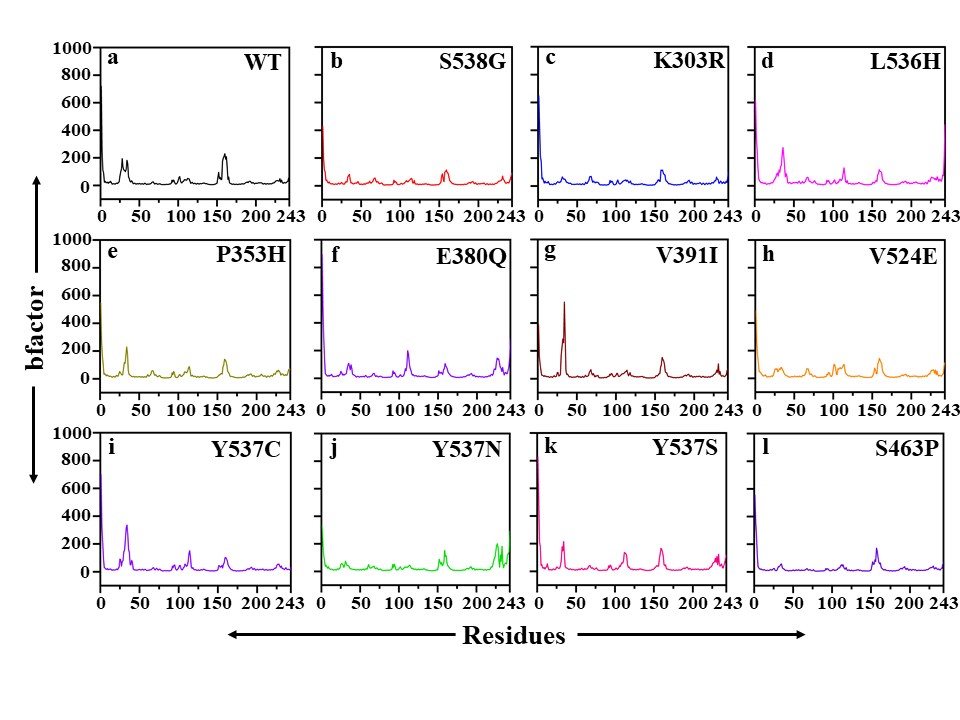


**Figure S3:** Showing the B-factor of all the apo systems (Wild and Mutant).


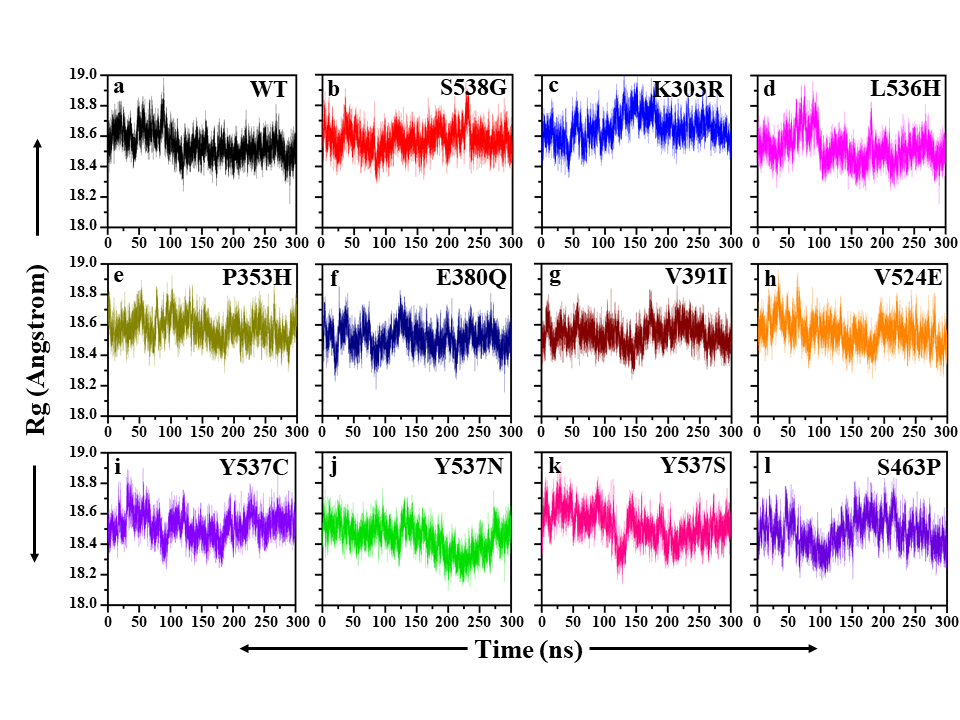


 **Figure S4:** Showing the Radius of gyration (ROG) of all the apo systems (Wild and Mutant).


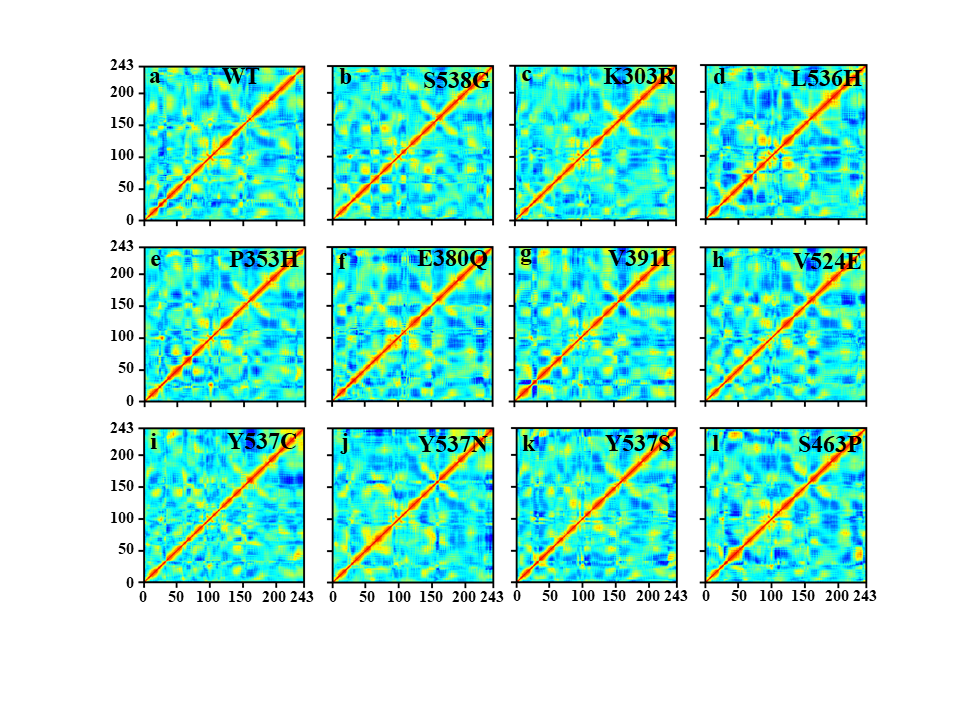


**Figure S5:** Showing the dynamics cross corelation motions of all the apo systems (Wild and Mutant).

| **Mutation** | **WT_SSE** | **WT_RSA (%)** | **WT_DEPTH (Å)** | **WT_OSP** | **WT_SS** | **WT_SN** | **WT_SO** | **MT_SSE** | **MT_RSA (%)** | **MT_DEPTH (Å)** | **MT_OSP** | **MT_SS** | **MT_SN** | **MT_SO** | **Predicted ΔΔG** | **Outcome** |
| --- | --- | --- | --- | --- | --- | --- | --- | --- | --- | --- | --- | --- | --- | --- | --- | --- |
| D538G | H | 103.0 | 3.2 | 0.18 | False | False | False | H | 109.2 | 3.5 | 0.28 | False | False | False | -0.17 | Reduced stability |
| E380Q | H | 35.4 | 4.0 | 0.37 | False | False | False | H | 38.5 | 4.1 | 0.38 | False | False | False | -0.4 | Reduced stability |
| L536H | t | 89.0 | 3.3 | 0.13 | False | False | False | t | 82.6 | 3.3 | 0.17 | True | False | False | -0.54 | Reduced stability |
| P535H | p | 37.9 | 3.8 | 0.36 | False | False | False | p | 70.8 | 3.5 | 0.2 | False | False | False | -0.39 | Reduced stability |
| S463P | b | 49.8 | 3.6 | 0.24 | True | True | False | b | 34.7 | 3.8 | 0.24 | False | False | False | -1.66 | Reduced stability |
| V392I | H | 0.0 | 9.0 | 0.54 | False | False | False | H | 0.0 | 9.1 | 0.6 | False | False | False | -0.11 | Reduced stability |
| H524E | H | 16.6 | 4.6 | 0.45 | False | False | True | H | 21.8 | 5.0 | 0.41 | True | False | False | 0.16 | Increased stability |
| Y537C | b | 28.7 | 4.3 | 0.42 | False | False | False | b | 24.6 | 3.9 | 0.36 | False | False | False | -0.09 | Reduced stability |
| Y537N | b | 28.7 | 4.3 | 0.42 | False | False | False | b | 25.7 | 4.2 | 0.37 | False | False | False | -1.1 | Reduced stability |
| Y537S | b | 28.7 | 4.3 | 0.42 | False | False | False | b | 26.7 | 3.9 | 0.36 | False | False | False | -0.77 | Reduced stability |
| K303R | p | 101.4 | 3.2 | 0.05 | False | False | False | p | 106.2 | 3.1 | 0.04 | False | False | False | 0.27 | Increased stability |

**Table S1:** Mutations results obtained to understand the effect of each residue change on the conformation of the protein.

**Table S2:** Hydrogen bonds their consistency and lengths are tabulated (before and after simulation).

| **Complex** | **Hydrogen bond** | | **Interacting Residue** |
| --- | --- | --- | --- |
|  | **Before MD(Å)** | **After MD(Å)** |  |
| **Wild** | 3.28 | 3.04 | Glu353 |
|  | 3.85 | n/a | Gly521 |
|  | 3.61 | 3.26 | Gly521 |
|  | 4.10 | 3.89 | His524 |
| **D538G** | n/a | n/a | Glu353 |
|  | n/a | n/a | Gly521 |
|  | n/a | n/a | His524 |
|  | 4.34 | 3.64 | Met421 |
| **E380Q** | n/a | n/a | Glu353 |
|  | 3.76 | 3.66 | Gly521 |
|  | 5.39 | n/a | His524 |
| **S463P** | n/a | n/a | Glu353 |
|  | 3.39 | 3.63 | Gly521 |
|  | n/a | n/a | His524 |
| **Y537C** | 5.41 | n/a | Arg394 |
|  | 6.91 | n/a | Glu353 |
|  | 2.96 | 3.98 | Gly521 |
| **Y537N** | n/a | 5.64 | His524 |
|  | n/a | 5.14 | Met421 |
| **Y537S** | 3.11 | n/a | Gly521 |
|  | 3.01 | n/a | Gly521 |
